# Factors influencing digital health literacy among older adults: a scoping review

**DOI:** 10.3389/fpubh.2024.1447747

**Published:** 2024-11-01

**Authors:** Zhen Shi, Xixi Du, Juan Li, Rongting Hou, Jingxuan Sun, Thammarat Marohabutr

**Affiliations:** ^1^College of Humanities and Education, Inner Mongolia Medical University, Hohhot, China; ^2^Faculty of Social Sciences and Humanities, Mahidol University, Nakhon Pathom, Thailand; ^3^School of Nursing, Hubei University of Medicine, Shiyan, Hubei, China; ^4^School of Ethnology and Sociology, Inner Mongolia University, Hohhot, China; ^5^Inner Mongolia University of Science and Technology, Baotou, China

**Keywords:** digital health literacy, older adults, influencing factors, online health information, e-HEALS, DHLI

## Abstract

**Background:**

The growth of digital technology, represented by the development of the Internet, has become popular among older adults. Implying digital health literacy on older adults also affects their ability to use digital technology to search, browse, understand, and evaluate health information to improve their health status. This scoping review aims to explore (1) the situation of digital health literacy among older adults and (2) the influencing factors on the digital health literacy of older adults.

**Methods:**

A scoping review was performed to evaluate evidence on influencing factors on digital health literacy among older adults in October 2023 employing data from literature indexed in PubMed, Web of Science, EBSCO, Springer Link, and CNKI with search terms such as “digital health literacy,” “e-health literacy,” “eHealth literacy” and “elderly people,” “aged people,” “old age.” The review comprised research articles that addressed issues related to digital health literacy and older adults, excluding non-research and research articles that only expressed opinions without concrete data or material support.

**Results:**

The final review included 28 articles from 4,706 retrieved records. The synthesis revealed that the digital health literacy of older adults was reflected in the scores of older adults in high-income countries, which were relatively high. In contrast, those in middle-income countries tended to be generally lower. The digital health literacy of older adults was affected by socio-demographic factors, related factors of electronic devices, and use and social support factors.

**Conclusion:**

Gaps of study discussed in this scoping review should be narrowed in further studies. Developing digital health literacy interventions with education and training programs should be considered to improve the digital health literacy of older adults. The digital divide among older adults should be bridged by improving social capital and family support through integrated intervention roles of government, community, and family.

## 1 Introduction

### 1.1 Background

The world has entered a digital age with the rapid development of digital technology through the Internet in the 21st century. Consequently, more individuals use digital technology to work, learn, and enjoy entertainment. Meanwhile, the world has also entered an era of population aging. In 2019, the number of individuals aged 60 or over was 1 billion, which will increase to 2.1 billion by 2050 (1). With the rapid growth of the older adult population, older adults are becoming a rapidly growing group of Internet digital technology users (2). For instance, in the United States, the percentage of adults aged 65 and older who own a smartphone increased by 24% from 18 to 42% between 2013 and 2017. In 2000, the percentage of older adults using the Internet was 14%, steadily increasing by 67% in 2017 (3). In China, with the development of science and technology, the number of Internet users reached 1.067 billion at the end of 2023 (4). Digital technology, represented by the growth of the Internet, has become popular in older adults’ daily lives, which may create new opportunities for healthy aging promotion in the era of population aging.

The application of digital technology in digital health development has been the focus of many countries recently. In February 2023, China issued the Overall Layout Plan for the Construction of Digital China, proposing to build an inclusive and convenient digital society and develop digital health (5). In 2022, the Digital Europe Health Commission aimed to promote the use of digital technologies in health by investing in the digital transformation of health systems and promoting the large-scale use of health technologies (6). The United Kingdom published the Digital Health and Social Care Plan, which identified the digital transformation of health and social care as its top priority (7). In South Korea, a pilot project using artificial intelligence and the internet to improve health care for older adults was launched (8). It was proposed that the rapid development of artificial intelligence, wearable devices, brain-computer interfaces, and other technologies has promoted the continuous iterative upgrading of digital health (9).

The application of digital technologies such as artificial intelligence, virtual reality, and machine learning has gradually changed the way older adults acquire and share health knowledge (10, 11). Internet use for health-related searches by older American adults increased from 24.8% in 2009 to 43.9% in 2018 (12). In China, the application of smart technology in the healthcare environment to meet the diverse health needs of older adults has become an important part of promoting healthy aging (13). Several studies have found that Internet use can improve the physical and mental health of older adults (14–16). A study ascertained that electronic information technology enhances healthy lifestyle behaviors (17). Overall, digital inclusion, which refers to the group on the application of the digital technology and adaptability (18), can help older adults better re-socialize because it can improve their digital literacy and lead them to adapt to the digital age’s lifestyle actively, significantly improving their quality of life and health (19). Electronic information technology is ubiquitous, providing resources and access to quality health information among older adults. Nonetheless, they still lag far behind younger individuals using electronic technology and the Internet (20). Older adults are disadvantaged in terms of physiological function, cognitive ability, social status, and economic status (21). The number of their digital devices, use skills, attitude toward new things, and the usefulness and ease of using the Internet and its related products and services have become essential factors in creating digital division among older adults (22). Imposing digital health literacy on older adults also affects their ability to use digital technology to search, browse, understand, and evaluate health information to improve their health status (23). In reality, older adults may have less experience in using modern media technologies and platforms in their social lives (24). As a result, this limits the ability of older adults to access, manage, and use health information to improve their health through using digital technologies such as the internet (25). Some older adults skilled at using technology are proficient in online searching as well as the creation and sharing of health information. Some of them have begun to use smart devices to monitor blood glucose (26). However, if older adults lack sufficient skills and knowledge in the use of e-health (27), and the quantity and quality of online health information are complicated and uneven, they may be harmed by false information on the internet if they cannot identify correct sources and facts (12).

Digital health literacy is an extension and expansion of the concept of electronic health literacy. Electronic health literacy, first developed by Norman and Skinner in 2006 (28), is defined as the ability “to seek, find, understand, and appraise health information from electronic sources and apply the knowledge gained to addressing or solving a health problem.” In 2011, Cameron Norman pointed out that the rapid shift in the informational landscape caused by Web 2.0 tools and environments suggests that it might be time to revisit the concept of eHealth Literacy (29). Since then, many scholars have redefined the concept of e-health literacy but still use the term eHealth literacy (30–34). In 2012, the concept of digital health literacy was first mentioned, and the concept of digital health literacy changed from focusing on the literacy skills of online information resources to emphasizing the interaction between individuals and the Internet (35). Scholars define it as the skills to search, select, appraise, and apply online health information and healthcare-related digital applications (36).

Many scholars found that older adults’ digital health literacy level was generally low using the eHEALS scale survey developed by Norman and Skinner in 2006. It is widely and frequently used to measure individuals’ e-health literacy worldwide (37). For example, Choi and DiNitto (38) surveyed 763 stay-at-home adults aged 60 years and older. They were found to have a lower digital health literacy overall, especially those older, those with lower socio-economic status, and had less computer use. A survey on the digital health literacy of 1,201 older adults in China found that the passing rate of digital health literacy among older adults was only 11.1% (39). The objective factors affecting older adults’ e-health literacy are Internet use, economic pressure, and education level. In contrast, the subjective factors are mainly older adults’ confidence, anxiety, and pressure in information technology (40, 41). Therefore, it is of great practical significance to sort out the situation and factors that influence digital health literacy among older adults. There have been three previous reviews on factors of digital health literacy in older adults. Among them, a scoping review reviewed the research progress of digital health literacy of older adults but only briefly listed the influential factors of digital health literacy of older adults without a detailed analysis of the specific impact (42). Another systematic review only sorted out the health literacy status of older adults and its influencing factors in China, with several pieces of literature that had insufficient representation (43). Another scoping review looked at factors promoting and hindering e-health use among older adults but did not focus strictly on digital health literacy (44). It is obvious that there is a lack of reviews on the factors involved in the digital health literacy of older adults. Therefore, a further review is essential. The unique contributions and practical significance of this scoping review include the following dimensions. Firstly, it provides a comparative perspective of the digital health literacy of older adults in different countries and regions. Secondly, it provides an analysis of the knowledge gaps on the influencing factors affecting the digital health literacy of older adults. Lastly, it supplies information which can be used as the foundation for recommendations to increase digital health literacy among older adults.

### 1.2 Research questions for scoping review

Digital health literacy of older adults can significantly affect their health and quality of life (45). However, digital health literacy among older adults could be different and influenced by related factors. This scoping review focuses on digital health literacy among older adults worldwide and various factors affecting their digital health to provide information and a basis for recommendations to promote digital health literacy for older adults. Therefore, the questions of the scoping review are: (1) What is the situation of digital health literacy among older adults? and (2) What factors influence older adults’ digital health literacy?

## 2 Methods

The methodology of this scoping review followed the framework outlined by Arksey and O’Malley (46) and Levac et al. (47), comprising five stages: identifying the research question, identifying relevant studies, study selection, charting the data and summarizing and reporting the results to describe the situation of digital health literacy among older adults and report influencing factors on their digital health literacy.

### 2.1 Data sources and search strategy

Literature in the form of research articles printed in English and Chinese related to older adults’ digital health literacy in academic databases was searched. The search in October 2023 only applied to all research articles published within the last ten years between 2014 and 2023.

Databases, including PubMed, Web of Science, EBSCO, and Springer Link, were the databases in which the literature published in the English language was searched. Several search keywords included terms related to digital health literacy and older adults. Four common terms related to digital health literacy, namely “digital health literacy,” “e-health literacy,” “eHealth literacy,” and “electronic health literacy,” were used as search terms. Subsequently, all search terms related to older adults, including “old,” “old people,” “older,” “older people,” “older adult,” “elder,” “elder people,” “elders,” “elderly people,” “elder adult,” “aged,” “aged people,” “aged person,” “aging” and “senior,” in combination with the four digital health literacy-related search terms were composed for Boolean search. A combination of these search terms was as follows: (“digital health literacy” OR “e-health literacy” OR “eHealth literacy” OR “electronic health literacy”) AND (“old” OR “old people” OR “older” OR “older people” OR “older adult” OR “elder” OR “elder people” OR “elders” OR “elderly people” OR “elder adult” OR “aged” OR “aged people” OR “aged person” OR “aging” OR “senior”).

The search for Chinese-language literature was conducted through the Chinese National Knowledge Infrastructure (CNKI) database. The CNKI is a network publishing platform integrating periodicals and magazines, doctoral theses, master theses, conference papers, newspapers, reference books, yearbooks, patents, standards, sinology, and overseas literature resources. It is the most comprehensive and widely used academic database in China. Additionally, the Chinese Social Science Citation Index and Peking University Core Journals Index, also widely recognized by Chinese scholars, were chosen as databases for searching. Two search terms related to digital health literacy, namely “digital health literacy” and “e-health literacy,” were used. The search also used three terms related to older adults, including “elderly people,” “old age,” and “old man.” The following combinations were used for searching for research articles in Chinese: “elderly people” AND “digital health literacy”; “elderly people” AND “e-health literacy”; “old age” AND “digital health literacy”; “old age” AND “e-health literacy”; “old man” AND “digital health literacy”; “old man” AND “e-health literacy.”

### 2.2 Study selection criteria

Literature on digital health literacy in older adults and studies on influencing factors are included in this scoping review. Research articles that meet the following inclusion criteria were selected for the review.

Inclusion criteria:

1. English and Chinese-language research articles published between 2014 and 2023;
2. The research participants were older adults aged 60 and above, or the average age of participants was 60 years or older, or more than 50% of the participants were 60 years old or older;
3. The research articles focusing on digital health literacy or electronic health literacy;
4. Empirical research on the situation and influencing factors of digital or electronic health literacy (quantitative, qualitative, or mixed methods).

Exclusion criteria:

1. Literature not written in the English or Chinese language;
2. Literature in the form of reviews, books, letters to the editor, and abstracts of speeches, Master’s theses, PhD dissertations and conference presentations;
3. Studies that did not aim at studying older adults, or the majority of the research population were not older adults;
4. Research articles only expressed opinions without concrete data or material support.

### 2.3 Data extraction and synthesis

After searching for literature, data gained from the searched research articles were extracted and then synthesized. Tables generated from the Microsoft Excel program were formed to illustrate data extraction and synthesis. The extracted data included the first author and year of publication, study location, study design, study setting, study sample, study population, period, digital health literacy measurement, digital health literacy score, and influencing factors. The included research results were summarized using descriptive syntheses showing the characteristics of included studies and the situation of digital health literacy among older adults in Table 1. The results of the influencing factors on the digital health literacy of older adults are presented in Tables 2–4.

**Table 1:** Characteristics of the included studies.

Author and year of publication	Location	Study design	Study setting	N	Study population (Age)	Period	DHL measurement	DHL score
Arcury et al. (40)	USA	Cross sectional study	In-person	106	Older adults (age 60 or older, 72.5%)	November 2014 to May 2016	8-item eHEALS (total score range = 8–40)	Total mean score 28.4
Berkowsky (48)	USA	Cross sectional study	Online	237	older adults aged 65+ (mean = 72.73)	2020	8-item eHEALS (total score range = 8–40)	Total mean score 24.5
Cajita et al. (49)	USA	Cross sectional study	In-person & online	129	Older adults had a history of heart failure (mean = 71.3)	February to August 2016	8-item eHEALS (total score range = 8–40)	Total mean score 27.3
Cao et al. (58)	China	Cross sectional study	In-person	4,218	Residents and dwelling in the community (mean = 71.9)	November and December 2020	The Chinese version of eHEALS This scale consists of three dimensions and eight items.	Total mean score 12.57
Cherid et al. (64)	Canada	Cross sectional study	In-person	261	Older with a recent fracture (age 65 or older, 59%)	September 2017 to March 2018	8-item eHEALS (total score range = 8–40)	Total mean score 65–74 years = 29 ≥ 75 years = 24
Choi and DiNitto (38)	USA	Cross sectional study	In-person and Telephone	980	Low-income, homebound older adults (mean = 71.31)	November 2012 to February 2013	8-item eHEALS (total score range = 8–40)	Average score 3.22 Total mean score 25.76
Cui et al. (59)	China	Cross sectional study	In-person	1,201	Aged over 60 (mean = 70.12)	January to February 2019	8-item eHEALS (total score range = 8–40)	Total mean score 17.24
Hoogland et al. (50)	USA	Cross sectional study	In-person	198	Older adults with cancer (age 65 or older, 51%)	July 2018 and September 2018	8-item eHEALS (total score range = 8–40)	Average score 3.44 Total mean score 27.52
Hu et al. (54) In Chinese	China	Cross sectional study	In-person	235	Older adult patients with chronic diseases (aged 60 or older)	March to August 2022	8-item eHEALS (total score range = 8–40)	Total mean score 22.11
Lee et al. (41)	USA and South Korea	Cross sectional study	In-person	217	community-dwelling respondents aged between 65 and 97(US: mean = 73, Korean: Mean = 71.34)	/	8-item eHEALS (total score range = 8–40)	US: Average score 2.70 Total mean score 21.6 Koreans: average score 3.56 Total mean score 28.48
Li et al. (60)	China	Cross sectional study	In-person	2,144	Older adults (mean = 72.01)	March to May 2021	8-item eHEALS (total score range = 8–40)	Total mean score 17.56
Li et al. (61)	China	Cross sectional study	In-person	2,300	Adults aged 60 or older (mean = 70.3)	June to August 2020	8-item eHEALS (total score range = 8–40)	Total mean score 18.6
Li et al. (39) In Chinese	China	Cross sectional study	In-person	1,201	Older adults range from 60 to 97 (median = 69)	January to March 2019	8-item eHEALS (total score range = 8–40)	Not report the mean eHEALS score
Liu et al. (62)	China	mixed-methods study	In-person & Online	337	Older adults in the context of the COVID-19 pandemic (Aged 60 or older)	June 2020 to February 2021	The eHealth Literacy Questionnaire (EHLQ) (comprises 15 items and four dimensions; Total scores range from 15 to 60)	Total mean score 46.47
Liu et al. (63)	China	Cross sectional study	In-person	572	Community-dwelling older adults ≥65 years (mean = 70.93)	September 2020 to April 2021	Digital health literacy assessment scale (the total score ranging from 15 to 75)	Total mean score 37.10
Liu et al. (55) In Chinese	China	Cross sectional study	In-person	472	Older adults (aged 60 or older)	January to March 2019	8-item eHEALS (total score range = 8–40)	Total mean score 13.76, SD = 7.30
Milne et al. (65)	Canada	Cross sectional study	In-person	83	Survivors of primary lung cancer (median = 71)	August 2013 to February 2014	8-item eHEALS (total score range = 8–40)	Total mean score 24
Price-Haywood et al. (51)	USA	Cross sectional study	Online	247	Older adults use patient portals (users: Mean = 63.4, Non-users: Mean = 65.2)	August 2015 and January 2016	8-item eHEALS (total score range = 8–40)	Total mean score user:32.9, SD = 4.7 Nonuser:24.7, SD = 8.0
Richtering et al. (69)	Australia	Cross sectional study	In-person	453	Population with moderate-to-high cardiovascular risk (mean = 67)	/	8-item eHEALS (total score range = 8–40)	Total mean score 27.2
Rojanasumapong et al. (67)	Thailand	Cross sectional study	In-person	110	Older adult patients with a history of diagnosed hypertension (mean = 67)	January 2016 and March 2016	8-item eHEALS (total score range = 8–40)	Total mean score 29.6, SD = 4.15
Schrauben et al. (52)	USA	Cross sectional study	In-person	932	Older adults with chronic kidney disease (CKD) (mean = 68)	2013 to 2015	8-item eHEALS (total score range = 8–40)	Not report the mean eHEALS score. Score ≥ 32 for adequate eHealth literacy; 27.2% of individuals with CKD have adequate eHealth literacy.
Tennant et al. (53)	USA	Cross sectional study	Telephone surveys	283	Baby boomers and older adults (mean = 67.46)	Feb-13	8-item eHEALS (total score range = 8–40)	Total mean score Users: 30.38, SD = 5.45 non-users: 28.31, SD = 5.79
Ubolwan et al. (68)	Thailand	Cross sectional study	In-person	1,237	Older adults using social media or the internet (mean = 66.9)	July to December 2016	8-item eHEALS (total score range = 8–40)	Total mean score 18.94, SD = 9.79
Vitolo et al. (70)	Italy	Cross sectional study	In-person	300	After COVID-19 outbreak among patients with frail and non-frail cardiology conditions (Median = 75,66–84)	March to September 2022	Digital Health Literacy Instrument (DHLI)	Total mean score 48.58, SD = 24.16
Yang et al. (71)	South Korea	secondary data analysis	In-person	187	Older adults (mean = 73.2)	November 2017 to February 2018	8-item eHEALS (total score range = 8–40)	Total mean score 30.95, SD = 4.17
Zhang et al. (56) In Chinese	China	Cross sectional study	In-person	915	Older adults (aged 60 or older)	September 2020 to April 2021	8-item eHEALS (total score range = 8–40)	Total mean score 22. 81
Zhou and Zheng (57) In Chinese	China	Cross sectional study	In-person	228	96*8/	June to September 2017	8-item eHEALS (total score range = 8–40)	Average score 1.51 Total mean score 12.08
Zibrik et al. (66)	Canada	case study	In-person	896	Older adults who are established immigrants who have lived in Canada for over 10 years (age 60 or older years, 75.5%)	2013 and 2014	8-item eHEALS (total score range = 8–40)	Total mean score 21.7

**Table 2:** Key and specific findings regarding socio-demographic factors influencing digital health literacy.

Key findings	Specific findings	Articles
Socio-demographic factors		
Age	Obstacle	(51, 58, 63)
	Greater age	(50, 52, 53, 55, 66)
	Lower age group	(38, 61, 64)
Sex	Older adult men	(54)
	Older adult men with more online time and social media habits	(61, 67)
	Rural older adult men	(55)
	Older adult men and women from migrant ethnic groups	(66)
Place of residence	Urban older adults	(39)
	Closer to urban area	(54)
	Province	(62)
Health-related factors	Implication of health status	(56, 63, 67)
	Risk of poor self-rated health status	(39)
	Existing health problems	(62)
	Depression	(38)
	Self-rated life stress	(39)
	Confidence in the management of chronic diseases	(52)
	Easiness and ability in health information	(60, 69)
	Factors of risk perception and health concept	(56, 63)
	Influence of access to health insurance and availability of community health services	(39, 56)
Socio-economic factors	Socio-economic status	(41, 57)
	Education	(39, 41, 48, 51, 53, 56, 63–68)
	Marital status	(55, 63)
	Income	(52, 54, 56, 66, 67)

**Table 3:** Key and specific findings on digital device-related factors influencing digital health literacy.

Key findings	Specific findings	Articles
Digital device-related factors		
Accessibility of Internet	Lack of access, usability challenges, and attitudes about technology	(66)
	Number of owned electronic devices and time spent online	(40, 53, 67)
	Frequency of Internet usage	(38, 48, 63)
	Frequency of searching for health information	(54, 56)
	Online time	(69)
	Duration of Internet usage and time spent per day	(63)
	The breadth of online activity	(48)
	Digital skills	(60)
	Patient portal use	(51)
Attitude toward the Internet and perceptions of online health information	Interest in using the Internet or smart devices	(51)
	Not feeling pressured to use a computer	(40)
	Feeling easy to use computer	(49, 63)
	Confidence with current digital skills	(41)
	Attitude toward online health information	(71)
	Information self-efficacy	(54)
	Credibility and reliability perception of online health information	(49, 57, 63)
	Risk perception of low online health information	(63)

**Table 4:** Key and specific findings on social support factors influencing digital health literacy.

Key findings	Specific findings	Articles
Social support factors		
Social capital of older adults	Social participation and social connection	(58)
	Structural and cognitive social capital	(59)
	Trust in primary healthcare providers	(49)
	Relying on physician knowledge for medical decisions	(40)
Support of family members	Family support	(54)
	Guidance from family members	(57, 63)
	Taking care of family members’ health and aging in the family	(39)
	English proficiency and cultural constraints	(66)

## 3 Results

**Figure 1:**
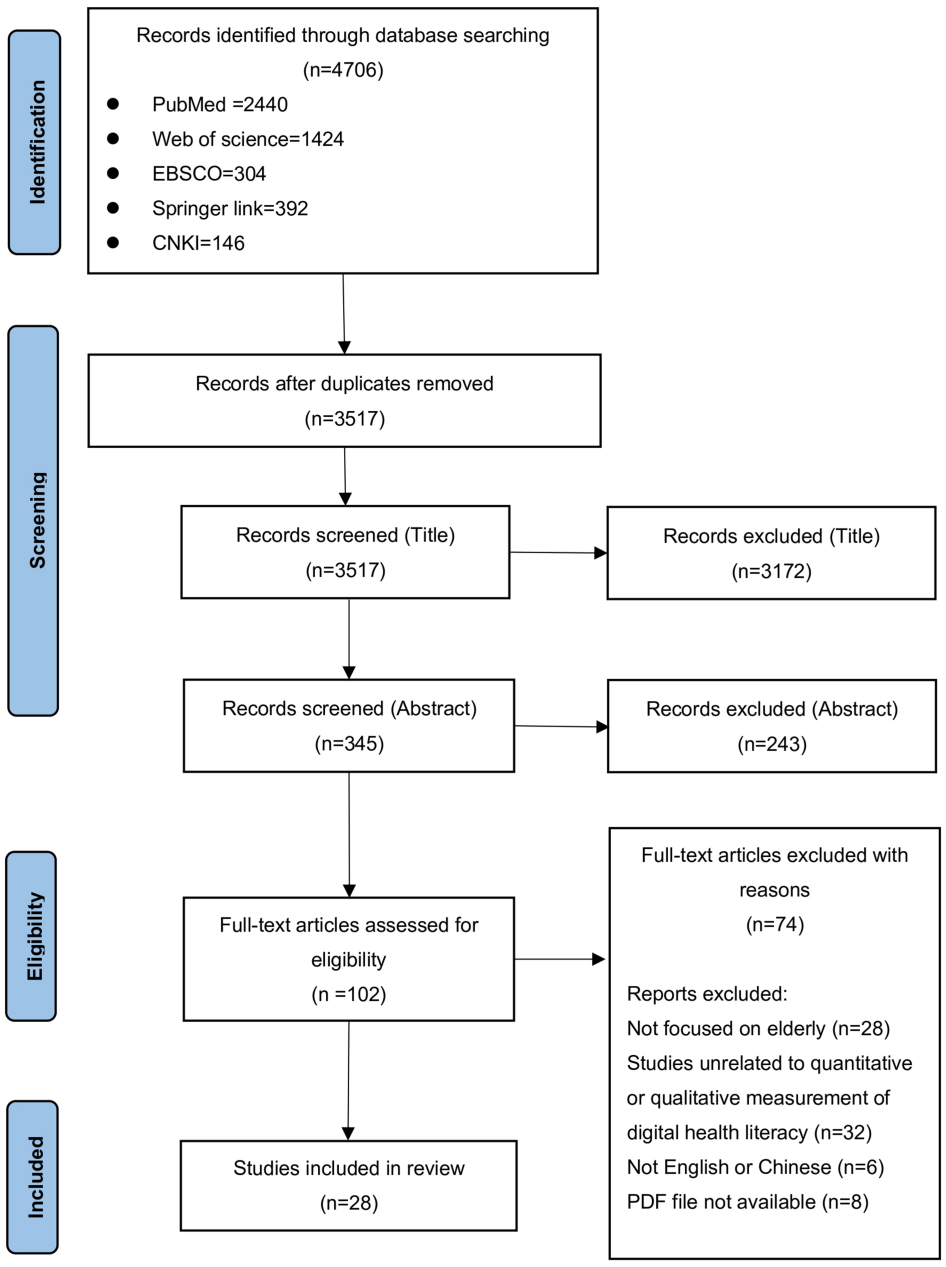
PRISMA flowchart of the literature search and study selection process.

The first step of literature screening was using keywords to search relevant research articles in the five databases. Thus, 4,706 articles were identified, including 2,440 articles from PubMed, 1,424 articles from Web of Science, 304 from EBSCO, 392 from Springer Link, and 146 from the CNKI. After removing duplicate articles, 3,517 articles were included. The following selection round was conducted based on title screening, and 3,172 articles were excluded. A total of 345 articles progressed to abstract screening, excluding 243 articles, and 102 articles were left. After reading the full text, it was found that 28 articles were not targeted at older adults; 32 articles did not use quantitative or qualitative data to carry out research; 6 articles were not published in English or Chinese; and 8 articles could not be found. Finally, 28 articles were included in the scoping review. A PRISMA flowchart of the literature search and study selection process is presented in Figure 1.

### 3.1 Characteristics of included studies

Table 1 summarizes the characteristics of the included studies. The data on the first author and year of publication, study location, study design, study setting, study sample, study population, period, digital health literacy measurement, and digital health literacy score are described as the characteristics of included articles in terms of language, location, research design, and participants, including measurement of digital health literacy.

#### 3.1.1 Language, location, research design, and participants

Five of the 28 research articles included were published in Chinese (17.86%), and 23 were published in English (82.14%). Survey respondents and data sources identified locations in the research articles. Eight articles were researched in the United States (38, 40, 48–53), whereas 11 articles were researched in China, among which five articles were published in Chinese (39, 54–57) and six published in English (58–63). Three researches were conducted in Canada (64–66), two in Thailand (67, 68), one each in Australia (69), Italy (70), and South Korea (71) and one in both the United States and South Korea (41).

Regarding research design, 25 articles used cross-sectional studies with first-hand survey data, which accounted for the majority (89.29%). One article also used a cross-sectional study, but the difference is that this article explicitly used secondary data for analysis (71). An article adopted a mixed research method (62) using quantitative questionnaires and qualitative semi-structured interviews. One article is a case study using focus group methods (66) for data collection. Regarding specific data collection methods, 22 articles adopted in-person data collection. One article adopted a telephone survey (53), while two adopted an online network and internal system data collection (48, 51). One article combines in-person and telephone data collection (38). Two articles used a combination of in-person and online data collection (49, 62).

Research participants identified in 28 articles were the older adults who met the inclusion criteria, among which 17 articles studied groups of older adults with specific conditions such as illnesses or in particular contexts. In 9 of 20 articles, older adults with illnesses were studied. Their health conditions included having a history of heart failure (49), recent fracture (64), cancer (50), chronic diseases (54), being survivors of primary lung cancer (65), being older adults with hypertension or diabetes, and use patient portals (51), having moderate-to-high cardiovascular risk (69), having a history of diagnosed hypertension (67), chronic kidney disease (CKD) (52) and being patients with frail and non-frail cardiology conditions after the coronavirus disease 2019 (COVID-19) pandemic (70). Six articles took older adults with specific backgrounds as research subjects. Their backgrounds were low-income, homebound older adults (38), older adults in the context of the COVID-19 pandemic (62), rural people aged 60 and above (55), baby boomers and older adults (53), older adults using social media or the Internet (68). British Columbia’s older Chinese and Punjabi immigrant adults are using eHealth for chronic disease self-management (66). Two articles categorize older adults as Internet users and non-users (51, 53). A total of 11 articles studied older adults in general as the research participants. For the distribution of research participants, the sample size of 22 articles was less than 1,000 individuals (78.57%). Only six articles had more than 1,000 respondents, of which five studies were conducted in China (39, 58–61) and one conducted in Thailand (68). One study conducted in Canada had the smallest sample size, with 83 participants (65), and the largest was a study conducted in China with 4,218 participants (58).

#### 3.1.2 Measurement of digital health literacy

Regarding digital health Literacy measurement tools, 24 articles adopted the 8-item eHealth Literacy Scale (eHEALS) developed by Norman and Skinner in 2006 (37). The total score of eHEALS ranges from 8 to 40, with higher scores indicating a higher level of digital health literacy. One article used the simplified five-point Likert Chinese version of eHEALS, which comprised three dimensions and eight items (58). One article used the eHealth Literacy Questionnaire (EHLQ), containing 15 items and four dimensions. Total scores ranging from 15 to 60 were used explicitly by Yang et al. (72) in 2021 to assess the digital health literacy of COVID-19-related participants. Higher scores were associated with a higher level of digital health literacy (62). One article used the digital health literacy assessment scale, developed by this Chinese research team through multiple steps, and it possesses high reliability and validity. This approach is specially used to measure the digital health literacy of older adults. The scale’s total score ranged from 15 to 75; that is, with a higher score, the level of digital health literacy is higher (63). One article used the Digital Health Literacy Instrument (DHLI) developed by van et al. in 2017. The DHLI explores seven digital skill categories measured by 21 self-report questions. Different from the set of scales mentioned above, the DHLI scale inquires about the difficulty of various tasks and the frequency of challenges encountered on the Internet, and higher scores indicate a lower level of digital health literacy (70).

For digital health literacy scores of the 24 articles using the eHEALS scale, 18 explicitly reported the total average score. Four articles reported the average score of eight items, whereas two articles did not report any form of the mean score but only used a score ≥ 32 as the cut-off point for adequate eHealth literacy (39, 52). The cut-off point of adequate eHealth literacy in different articles was inconsistent. The two articles mentioned above used 32 points as the cut-off point, which is different from 26 points (69), which are set as the cut-off point between high and low digital health literacy in other articles. The remaining four articles that did not use the eHEALS scale also reported the total average score of digital health literacy in older adults. However, it was clear that the total average score of these four articles could not be compared with those of other articles using the eHEALS scale.

### 3.2 Situation of digital health literacy among older adults

The data on digital health literacy scores are described as digital health literacy among older adults extracted in Table 1. Digital health literacy was measured using the eHEALS scale, and scores could be translated to digital health literacy levels. A total of 22 articles used the eHEALS scale, and average scores were calculated to determine older adults’ digital health literacy levels. These articles are distributed in different countries. Seven articles were researched in the United States, seven in China, three in Canada, two in Thailand, one in South Korea, one in Australia, and one in both the United States and South Korea.

The older adults in the United States had the highest score of digital health literacy of 32.9 (51), whereas the older adults in China had the lowest score of 12.08 (57). The scores indicated in seven articles on the digital health literacy of older adults in the United States were 28.4 (40), 24.5 (48), 27.3 (49), 25.76 (38), 27.52 (50), of patient portal users 32.9 and non-users 24.7 (51) and on Web 2.0 for health information users 30.38 and non-users 28.31 (53). The scores mentioned in seven articles on the digital health literacy of Chinese older adults were 17.24 (59), 22.11 (54), 17.56 (60), 18.6 (61), 13.76 (55), 22.81 (56), and 12.08 (57). The scores reported in three articles on the digital health literacy of Canadian seniors were 29 for the age group 65–74 years, 24 for the age group ≥75 years (64), 24 (65), and 21.7 (66). In two articles studied in Thailand, the digital health literacy scores of the older adults were 29.6 (67) and 18.94 (68), while that of the South Korean older adults reported in one article was 30.95 (71). One Australian article mentioned the score of digital health literacy among older adults was 27.2 (69). An article covering the United States and South Korea showed that older adult Americans’ digital health literacy score was 21.6, whereas that of older adults in South Korea was 28.48 (41).

### 3.3 Influencing factors on digital health literacy among older adults

The factors that influence the digital health literacy of older adults were divided into three dimensions. They are socio-demographic factors, as shown in Table 2, digital device-related factors in Table 3, and social support factors in Table 4.

#### 3.3.1 Socio-demographic factors

The socio-demographic factors included age, sex, place of residence, health-related factors, and socio-economic status. Table 2 shows vital and specific findings on socio-demographic factors as the influencing factors on digital health literacy. Age was an obstacle to the digital health literacy of older adults (51, 63, 68). Greater age denoted a lower level of digital health literacy (50, 52, 53, 55, 66). The digital health literacy of the lower age group of older adults was significantly higher than that of the greater age group. This might relate to the relatively low frequency of Internet and social media use (38, 64), as cognitive decline with age leads to a decline in the ability to learn and understand online health knowledge (61). There were sex differences in digital health literacy among older adults. Older adult men had higher levels of digital health literacy than older adult women (54). This might relate to more online time and social media habits of older adult men (61, 67). The level of digital health literacy of rural older adult men was also higher than that of rural older adult women (55). A study on immigration in British Columbia, Canada, found that sex factors varied inconsistently among different ethnocultural groups. In the Chinese immigrant group, men’s digital health literacy level was higher than women’s. In contrast, in the Punjabi immigrant group, women’s digital health literacy level was higher than men’s (66). For place of residence, the level of digital health literacy of urban older adults was significantly higher than that of rural older adults (39). In the older adult group with chronic diseases, the older adult patients who lived closer to the urban area had more comprehensive access to health information conducive to improving digital health literacy (54). Another online survey on the digital health literacy of older Chinese adults conducted between June 2020 and January 2021 found that the digital health literacy of older adults living in the Hubei Province of China in the previous month of the survey was significantly lower than that of older adults living in other provinces outside Hubei or abroad (62).

Regarding the relationship between health-related factors and the digital health literacy of older adults, the health status of older adults was positively correlated with digital health literacy. Notably, better health status implied higher digital health literacy (56, 63, 67). Poor self-rated health status was a risk factor for digital health literacy in older adults (39), as older adults with existing health problems had lower levels of digital health literacy (62). For example, older adults with a diagnosis of depression, although they used the Internet more often, had lower levels of digital health literacy due to lower self-evaluation (38). The older adults with higher self-rated life stress also had digital health literacy at lower levels (39). Higher confidence in managing chronic diseases also signified a higher level of digital health literacy (52). Additionally, older adults’ health literacy was highly correlated with digital health literacy. The higher the level of digital health literacy was, the easier it was to pay attention to health information through multiple channels. Moreover, the ability to identify and judge health information was thus improved in digital health literacy (60, 69). Both health risk perception and concepts are critical protective factors for their health; that is, more attention to health would positively impact the motivation to seek health information online, thereby improving their digital health literacy (56, 63). Furthermore, access to health insurance and the availability of community health services influenced digital health literacy (39, 56).

Under the realm of socio-demographic factors, socio-economic status includes education, marital status, and income. Overall, higher socio-economic status indicated a higher level of digital health literacy among older adults (57). Older adults with lower socio-economic status, such as those living in rural areas, receiving less education, being unmarried, and having a lower income, might have a lower level of digital health literacy (41). Expressly, higher education levels of older adults indicated higher levels of digital health literacy (41, 53, 56, 63, 67, 68). Lower education level was an obstacle to improving the digital health literacy of older adults (48, 66), especially the education level of primary school or below, which was a risk factor for digital health literacy (39). According to the influence of different education levels on the digital health literacy of older adults, the digital health literacy of older adults with education above high school level was significantly higher than that below high school (51). Some studies took college education as a distinction. The digital health literacy of the older adults who studied at college was significantly higher than that of the older adults who did not (64, 65). A higher level of digital health literacy among married older adults might relate to better family functioning and, thus, better family emotional support among married older adults (63) and married rural older adults (55). A higher income level denoted a higher level of digital health literacy in older adults (66). Personal income (67), monthly income (56), or annual income (52) had a significant positive impact on the digital health literacy. Meanwhile, for older adults with chronic diseases, their higher income meant a higher level of digital health literacy (54).

#### 3.3.2 Digital device-related factors

Digital device-related factors were reflected in the accessibility of the Internet, the attitude toward the Internet, and perceptions of online health information. Key and specific findings on aspects related to digital devices as the influencing factors on digital health literacy are presented in Table 3. Regarding access to the Internet, the use of the Internet and accessibility of electronic resources were significant factors affecting the digital health literacy of older adults (52, 65, 68, 73). Further, the digital device-related factors include the lack of access, usability challenges associated with aging, and attitudes toward technology (66). Notably, the number of owned electronic devices and the time spent online were closely related to the digital health literacy of older adults. A higher number of electronic devices that could access the Internet or search for health information denoted a higher level of digital health literacy (50, 53, 67). The frequency and time of Internet use also greatly affected the digital health literacy of older adults. Higher Internet usage frequency implied higher digital health literacy levels (38, 48, 63). Significantly, the higher frequency of searching for health information online indicated a better level of digital health literacy among older adults (54, 56). For rural older adults, a higher frequency of Internet usage signified a better level of digital health literacy (55). Furthermore, online time could increase their digital health literacy (69). The duration of Internet usage and time spent using the Internet per day could significantly affect the digital health literacy of older adults (63). The breadth of online activity among them also affected digital health literacy (48). Finally, digital skills were closely related to the digital health literacy of older adults. With more proficiency in Internet usage, the level of digital health literacy tended to be higher (60). Moreover, an article that studied the impact of the older adult patients’ portal use on digital health literacy found that the patient portal use could increase the digital health literacy of older adults (51).

Attitudes toward the Internet and perceptions of online health information greatly influenced the digital health literacy of older adults. Their interest in using the Internet or smart devices affected digital health literacy (51). In particular, older adults who did not feel pressured to use a computer (40) or found it relatively easy to use (49, 63) and were confident with their current digital skills (41) had relatively high digital health literacy. The attitude toward online health information also affects the digital health literacy of older adults (71). Information self-efficacy, that is, confidence in information search, comparison, and evaluation, could directly predict their digital health literacy (54). The credibility and reliability perception of online health information was a positive factor in subjectively searching for electronic resources and applying the obtained information to deal with and solve health problems (49, 57, 63). The risk perception of low online health information literacy would increase the enthusiasm of older adults for digital health services, thus improving their digital health literacy (63).

#### 3.3.3 Social support factors

Social support factors included older adults’ social capital and family members’ support. Table 4 summarizes vital and specific findings on social support factors influencing digital health literacy. A higher level of social capital in the dimensions of social participation, social connection, trust, and reliability implied a higher level of digital health literacy among older adults. For older adults aged 70–79 years, higher social participation denoted higher digital health literacy. Higher social connection levels signified higher digital health literacy among older adults aged 60–79 years. However, the study did not find a relationship between social support and digital health literacy (58). In contrast, an article found that structural social capital, such as social participation, social support, and connection, affected digital health literacy in older adults, while cognitive social capital, such as trust, cohesion, and reciprocity, did not (59). Trust in primary health care providers such as doctors and nurses affected older adults’ use of digital health resources (49). The older adults relying on physician knowledge for medical decisions had a higher level of digital health literacy (40).

Support from family members was a factor affecting digital health literacy as well. Family, friends, and society have a high degree of support and care for the older adults at the material, economic, and emotional levels. When older adults needed family support, their participation was conducive to improving their access to and use of digital resources (54). Family members taught older persons to use the Internet to find health information. The frequency of receiving guidance from family members significantly affected the digital health literacy of older adults (57, 63). Taking care of grandchildren’s health was a promotive factor for the digital health literacy of older adults as they could collect health-related information to look after their grandchildren and take care of themselves. Aging in the family might be a risk factor for the digital health literacy of older adults because of a reduction in social ties (39). A Canadian study on migrants found that limited English proficiency significantly restricted access to health care and e-health resources, affecting older adults’ digital health literacy. Cultural constraints, such as the value of filial piety, deteriorated the patience of children, grandchildren, and caregivers in helping older adults to learn how to search for information online, thus also affecting their digital health literacy (66).

## 4 Discussion

Most scholars still use the eHEALS scale to measure the digital health literacy of older adults. However, only a few scholars developed a scale of their own. The level of digital health literacy of older adults in the United States was the highest, while that of the older adults in China was the lowest. The possible reason is that China gained full Internet access in 1994, later than many developed countries, and the older adults population was even more marginalized by the Internet. In other countries, except one study in Thailand, older adults scored above 20 on digital health literacy. In particular, South Korean seniors scored relatively high on digital health literacy. This may be related to the value in modern Korean culture about the usefulness and importance of using the Internet in daily life (74). Moreover, the documents that were searched showed differences in the digital health literacy of older adults in different countries and within the same countries.

The factors that influence the digital health literacy of older adults were divided into three dimensions: socio-demographic factors, digital device-related factors, and social support factors. The socio-demographic factors included age, gender, place of residence, health-related status, and socio-economic status, including education, marital status, and income. Internet accessibility, attitudes toward the Internet, and online health information were the digital device-related factors. The social support factors comprised older adults’ social capital and family members’ support. Although the documents searched discussed the impact of socio-demographic factors and social support factors on the digital health literacy of older adults, digital device-related mediating factors were found to be very important. Some studies have shown that socio-demographic and social support factors may impact the digital health literacy of older adults and, in turn, the mastery of digital skills or the use of intelligent devices. Living with children and having good relationships with family members significantly affect their motivation to use digital technology (57, 75). The lack of social and family support for older adults and stereotypes of older adults seriously affect the promotion of digital media in this group (48). In short, a high level of social capital provides more learning and communication opportunities for older adults to a certain extent. It also has a positive impact on their use of electronic products to obtain and utilize health information and promote the formation of healthy behaviors (59). Older adults with better social and economic status are more likely to own more electronic devices. Older adults with greater levels of education are more likely to have more proficient information technology capabilities to improve their digital health literacy through education and training (53). Accordingly, underlying social structures, such as personal characteristics, social status, and social support, influence the digital health literacy of older adults as well as their individual motivation and effectiveness in using the internet or electronic resources for health purposes (59). However, the existing research on the factors that influence the digital health literacy of older adults still lacks effective theoretical perspectives and models, leading to strong subjective randomness in including influencing factors. Only a few articles explored the influencing factors of older adults’ digital health literacy from the perspective of social capital or based on the Anderson model of health service utilization, including three crucial components: predisposing, enabling, and need-for-care factors that either facilitate or impede patients’ use of services (76). It is concluded that existing interventions are neither theory-based nor use high-quality research design (73).

Regarding research methods, the existing research works on the digital health literacy of older adults mainly used a quantitative research approach based on cross-sectional data. They lacked using longitudinal data and a qualitative research approach. A lack of in-depth interpretation in quantitative research affected the depth and reliability of research to some extent. Therefore, in future studies, it is necessary to increase the use of longitudinal data to track temporal change characteristics of the digital health literacy of the older adults. Simultaneously, based on quantitative research, the qualitative research method should be added as a mixed-method to increase the complementary strength of research results. Additionally, more reliable methods should be further used to test the impact of influencing factors on the digital health literacy of older adults. The measurement of digital health literacy of older adults still lacked an effective scale suited to the current development of the Internet, which targeted explicitly for better accurate measurement of the digital health literacy of the older adults group. The digital health literacy of older adults is measured with the eHEALS scale. Although this scale has been translated into multiple languages and used with different populations to compare people in various settings, it is a self-reported scale criticized for representing self-efficacy rather than actual digital health literacy abilities (77). Furthermore, this scale is a tool for assessing the Web 1.0 skills of a large audience of passive readers. Nonetheless, it is unclear how accurately it can measure the use of Web 2.0 technologies widely employed due to their ability to read and write to find and evaluate health information (29, 78). Although several studies have developed a new digital health literacy scale for older adults that is in line with the current development of the Internet, the recognition and promotion of the new scale are not sufficient. Therefore, a more comprehensive tool is needed to measure older adults’ ability to use digital health through various applications. In future research, it is necessary to develop a high reliability and validity scale that scholars widely accept and effectively apply to the older adult population. The existing research on the influencing factors of digital health literacy of older adults lacked the exploration of the correlation or mediating role among the influencing factors. They mainly discussed the factors influencing older adults’ digital health literacy in different dimensions and levels. However, various influencing factors may interact, possibly affecting older adults’ digital health literacy. However, they have not yet discussed the correlation or mediating role among the influencing factors.

## 5 Limitation

Per the procedure of scoping review, this study conducted a literature search on five commonly used databases and concluded a total of 28 research articles. It has yielded considerable results on digital health literacy among older adults and the factors influencing their digital health literacy. However, studies on the digital health literacy of older adults in other databases were not included. Several articles on older adults’ digital health literacy may be missed. All articles included have only been published in English and Chinese. Articles published in other languages are excluded, which may lead to incomplete literature retrieval.

## 6 Conclusion

Through the scoping review on the influencing factors of digital health literacy of older adults, the situation was reflected in regional and intra-group differences between countries in terms of their digital health literacy level. The scores of digital health literacy of older adults in the United States and other high-income countries, including Canada, South Korea, Australia, and Italy, were relatively high. In contrast, those in middle-income countries like China and Thailand tended to be generally lower. Furthermore, older adults in rural areas had a lower digital health literacy than those in urban areas. Regarding the influencing factors, the digital health literacy of older adults was affected by socio-demographic factors, related factors of electronic devices and use, and social support factors. Higher socio-economic status, more extensive accessibility of electronic devices and the ability to use them, and more excellent social support mean a higher level of digital literacy among older adults. However, there are existing gaps of study discussed in this scoping review, including the lack of effective theoretical perspectives and models, the lack of longitudinal data and qualitative research approach, the lack of in-depth interpretation in quantitative research, the lack of effective scale suited to current development of the Internet targeted explicitly at better accurate measurement of the digital health literacy of the older adults group, the lack of recognition and promotion of recently developed scale and the lack of exploration of the correlation or mediating role among the influencing factors. These gaps should be narrowed in further studies.

Developing digital health literacy interventions should fully consider the local socio-cultural context to improve the digital health literacy of older adults. With a strengthening focus on older adults, especially those in rural areas with poor health and low socio-economic status. Education and training programs should be tailored to the needs of older persons with different socio-demographic characteristics to ensure their effectiveness. As Internet accessibility and attitudes towards the Internet and online health information are primary conditions and critical elements of the digital health literacy for older adults, the digital divide among older adults should be bridged to improve their digital skills and mitigate health information discrimination. Social capital and family support should be enhanced by expanding social participation and building social support networks for older adults with integrated government, community, and family intervention roles.

## Data Availability

The data that support the findings of this study are available from the corresponding author upon reasonable request.
